# Quality assessment of dry eye educational videos on Xiaohongshu and differences by uploader type

**DOI:** 10.3389/fmed.2026.1868829

**Published:** 2026-06-03

**Authors:** Shiyang Niu, Bing Han, Xingyu Liu, Hui He, Yajun Wu, Dawei Sun, Lijun Qu

**Affiliations:** 1 Department of Ophthalmology, The Second Affiliated Hospital of Harbin Medical University, Harbin, China; 2 Graduate Education Department, The Second Affiliated Hospital of Harbin Medical University, Harbin, China; 3 Clinical Skills Training Center, The Second Affiliated Hospital of Harbin Medical University, Harbin, China; 4 The Second Affiliated Hospital of Harbin Medical University, Harbin, China

**Keywords:** dry eye, health information quality, patient education, short videos, Xiaohongshu

## Abstract

**Background:**

Xiaohongshu has become an important platform for the public to obtain eye health information, but the quality and uploader-related differences of dry eye educational videos remain unclear.

**Methods:**

In this cross-sectional study, Xiaohongshu’s “Video” section was searched on April 16, 2026, using the Chinese keyword “干眼” (dry eye). The first 200 videos generated by the default sorting algorithm were screened. After excluding duplicate, promotional, off-topic, and reposted content, 136 videos were included. As all eligible videos were uploaded by individual accounts, comparisons were performed between non-medical individual users and individual medical users. Video characteristics, content coverage, and engagement metrics were extracted, and video quality was assessed using DISCERN, PEMAT-A/V, and the Global Quality Score.

**Results:**

Among the 136 included videos, 108 were uploaded by non-medical individual users and 28 by individual medical users. Video content was mainly concentrated on lifestyle recommendations (87.5%) and treatment or management strategies (62.5%), whereas topics such as definition, diagnosis, classification, and follow-up were less frequently covered. Compared with videos uploaded by individual medical users, those uploaded by non-medical individual users had a higher prevalence of incorrect or potentially misleading information (57.4% vs. 10.7%; OR = 11.23, 95% CI: 3.20–39.47). In addition, videos uploaded by individual medical users scored significantly higher than those from non-medical individual users in DISCERN total score, PEMAT understandability, PEMAT actionability, and GQS score, with all comparisons reaching statistical significance (all *p* < 0.001). Correlation analysis indicated that the number of covered content domains was positively associated with the DISCERN total score, GQS score, and PEMAT understandability.

**Conclusion:**

Dry eye-related videos on Xiaohongshu varied substantially in quality and mainly emphasized lifestyle recommendations and treatment management, with limited coverage of systematic medical knowledge. In this cross-sectional sample, videos uploaded by individual medical users were associated with higher quality scores, broader content coverage, and a lower prevalence of incorrect or potentially misleading information than videos from non-medical individual users. These findings suggest the potential value of platform-level quality control, improved mechanisms for identifying and recommending professional content, and greater participation of ophthalmic healthcare professionals in standardized science communication.

## 1 Introduction

Dry eye is a multifactorial chronic ocular surface disease characterized by loss of tear film homeostasis and may be accompanied by ocular discomfort, fluctuating visual function, ocular surface inflammation, and ocular surface damage (1–7). Previous studies have shown that dry eye imposes a substantial disease burden, compromising vision-related quality of life and interfering with reading, digital screen use, and daily work productivity (8, 9). As the use of video display terminals continues to increase, growing attention has been paid to the association of video display terminal work and digital eye strain with dry eye-related symptoms (10–13). Therefore, the provision of accurate, understandable, and actionable dry eye-related health information has become an important component of ocular surface disease prevention and management.

The Internet and social media have become major channels for the public to access health information. Systematic reviews have shown that although online health information is highly accessible and rapidly disseminated, it often remains limited in source transparency, evidentiary support, content completeness, and scientific accuracy. In social media environments, misinformation, commercially driven messaging, and oversimplified narratives may spread more readily, potentially influencing public understanding of disease, healthcare-seeking decisions, and self-management behaviors (14–19). Accordingly, systematic evaluation of the quality, potential risks, and dissemination characteristics of social media-based health information has become an important focus of digital health research.

Xiaohongshu, also known in some international contexts as RedNote, has a distinct platform ecology compared with other short-video and social media platforms. Its content environment is strongly shaped by lifestyle-oriented narratives, consumer experience sharing, and product-use recommendations. In health-related contexts, this ecology may increase the visibility of personal experiences and product-oriented advice. Dry eye is particularly relevant in this setting because its prevention and self-management are closely linked to daily visual habits, screen exposure, artificial tears, warm compresses, eyelid massage, and eye-care products. Therefore, dry eye-related content on Xiaohongshu may combine personal experience narratives, commercial messages, and health advice, creating a platform-specific context in which incomplete or potentially misleading information may be disseminated.

In recent years, there has been a marked increase in studies evaluating the quality of disease-related educational content on Chinese short-video platforms. Studies addressing topics such as dry eye, stroke, pediatric influenza vaccination, Crohn’s disease, and thyroid-associated ophthalmopathy have consistently suggested that short-video platforms offer clear advantages for health information dissemination, while substantial limitations persist in content completeness, reliability, and quality differences across uploader types (20–26). Similar issues have been reported in studies of short videos related to certain cancers and gastrointestinal diseases, indicating that variability in the quality of health-related short-video information may be a common phenomenon (27–30). Previous research suggests that platform ecosystems and uploader profiles may influence the scientific accuracy, understandability, and dissemination performance of health-related videos. To date, systematic single-platform studies of dry eye educational videos on Xiaohongshu remain limited, and comprehensive assessments of uploader-related differences, content coverage, potentially misleading information, and engagement characteristics are especially lacking. Against this background, the present study focused on the “Video” section of Xiaohongshu and evaluated dry eye-related videos retrieved using the Chinese keyword “干眼” (dry eye). By combining general quality assessment tools with a dry eye-specific content and incorrect or potentially misleading information coding framework, we aimed to characterize the quality, completeness, understandability, actionability, and potential information risks of dry eye-related videos in this experience-sharing platform environment. We further compared videos uploaded by individual medical users and non-medical individual users to explore how uploader identity may be associated with content coverage, incorrect or potentially misleading information, and educational quality.

## 2 Materials and methods

### 2.1 Study design and ethical considerations

This cross-sectional study analyzed publicly available video content on Xiaohongshu and did not involve participant recruitment, intervention, direct interaction with users, or identifiable private information. Therefore, ethics review and informed consent were not required according to institutional requirements. All data were accessed and analyzed in accordance with Xiaohongshu’s publicly available terms of use, which were accessed on April 16, 2026, and relevant institutional requirements. Throughout the study, we only observed, recorded, and analyzed publicly displayed information, without interacting with uploaders or users.

### 2.2 Search strategy and video screening

On April 16, 2026, we searched the “Video” section of Xiaohongshu using the Chinese keyword “干眼” (dry eye) under the platform’s default sorting algorithm, and consecutively recorded the first 200 results as the initial sample. The keyword “干眼” was selected because it is a broad and commonly used Chinese lay term corresponding to dry eye and was intended to approximate a common user search pathway on the platform. To minimize prior personalization, the search was performed using a newly registered account created with a mobile phone number that had not previously been used for Xiaohongshu registration. The account had no prior browsing history, search history, saved content, liked content, followed accounts, or dry eye-related interaction records before the search. The search was conducted at the Second Affiliated Hospital of Harbin Medical University, Harbin, China, using a 13-inch MacBook Air with an Apple M4 chip and 16 GB memory running macOS Tahoe version 26.4. No manual adjustments were made to the platform’s personalization or recommendation settings. No additional filters were applied, and the platform’s default search and sorting settings were retained to approximate a common user search pathway. Because the platform uses a proprietary and dynamic ranking algorithm, residual influences from geographic location, device environment, search time, and platform-level recommendation mechanisms could not be fully excluded. Repeated searches were not performed.

Videos were included if they were related to dry eye, presented in Chinese, playable, and originally uploaded. Videos unrelated to dry eye, promotional videos, reposted content, and duplicate videos were excluded from the main quality assessment. Promotional videos were defined according to the primary purpose of the video. Videos were classified as promotional when their main purpose was to advertise, sell, or direct viewers to purchase a specific product or service, such as videos centered on product efficacy claims, brand promotion, purchase links, sales guidance, or service marketing. By contrast, videos were retained when their main content was dry eye-related education or personal experience sharing, even if they mentioned product categories, specific brands, or product-use experiences. These retained videos were further coded for product recommendation, brand visibility, and commercial bias.

Promotional videos were excluded from the main quality assessment because DISCERN, PEMAT-A/V, and GQS were used to evaluate health educational materials, whereas videos primarily designed for advertising or product sales do not necessarily aim to provide general patient education. However, because promotional content is part of the information environment encountered by ordinary users on Xiaohongshu, promotional videos were retained for a predefined secondary analysis comparing video duration and engagement metrics between included educational/experience-sharing videos and promotional videos. Two researchers independently screened all candidate videos according to these criteria, and disagreements were resolved through discussion or adjudication by a third researcher.

### 2.3 Data extraction and uploader classification

For each included video, we extracted video duration, number of likes, comments, saves, and shares, publication date, and the number of days between publication and the search date. Uploader type, account verification status, and video presentation characteristics were also recorded using publicly available information from the video page and uploader profile. In the final sample, all eligible videos were uploaded by individual accounts; content from for-profit organizations or institutions was mainly excluded during screening because of its promotional nature, and the formal comparison was therefore limited to individual medical users and non-medical individual users.

Uploader classification was performed according to a predefined coding rule based on publicly available account information and video content. Individual medical users were defined as individual accounts with clearly displayed platform-based medical professional qualification verification on the account profile or clear evidence of medical professional identity based on publicly available account information and video content. Evidence for medical professional identity included platform-based medical professional qualification verification, certified professional category, affiliated institution, professional title or position when available, doctor appearance on camera supported by other publicly available profile information, or consistent provision of specialty-specific medical explanations. Non-medical individual users were defined as individual accounts without sufficient evidence of medical professional identity. Accounts mainly presenting patient experience, lifestyle sharing, personal product-use experience, or general health-related content were classified as non-medical individual users when sufficient evidence of medical professional identity was not available. Doctor appearance on camera, self-reported medical identity, or health-related content alone was not considered sufficient evidence unless supported by other publicly available profile information or consistent specialty-specific medical explanations.

Two reviewers independently classified uploader type according to this predefined rule. Inter-rater agreement for uploader classification was assessed using Cohen’s kappa. Disagreements were resolved through discussion or adjudication by a third reviewer. Detailed classification criteria are provided in Supplementary Table S1.

The variables “verified account,” “doctor appearing on camera,” “product recommendation,” and “brand visibility” were coded as binary variables: accounts with an official platform verification label were coded as verified accounts; videos in which a doctor appeared on camera and provided an explanation were coded as doctor appearing on camera; videos that explicitly recommended a product category or guided use or purchase were coded as product recommendation; and videos showing a specific brand name, trademark, packaging, or clear brand indication were coded as brand visibility.

### 2.4 Video content coding and assessment of incorrect or potentially misleading information

The video content evaluation framework was developed with reference to TFOS DEWS II, the Asia Dry Eye Society consensus, and the content coding approaches used in previous studies of dry eye-related short videos (1–6, 20). According to the observed content characteristics of the sample, video content was categorized into eight domains: definition, classification, symptoms, risk factors, diagnosis, treatment and management, lifestyle recommendations, and follow-up. Each domain was coded in a binary manner, with 1 indicating that the domain was mentioned and 0 indicating that it was not mentioned, and the total number of covered domains was calculated for each video. Lifestyle recommendations mainly included advice on visual habits, environmental modification, warm compresses, sleep, screen exposure, and daily care, whereas treatment and management included artificial tears, medications, physical therapies, and healthcare-seeking guidance. Content coding was independently performed by two researchers with ophthalmology backgrounds, and the final coding database was established after review. Discrepancies in content coding were resolved through discussion, and unresolved items were adjudicated by a third researcher.

The presence of “incorrect or potentially misleading information” was assessed according to current dry eye consensus statements and clinical frameworks. Detailed operational criteria and judgment boundaries for each category are provided in Supplementary Table S2. Videos with primarily promotional purposes were excluded from the main quality assessment, whereas included educational or experience-sharing videos were still coded for commercial bias when product-oriented claims or recommendations were embedded within otherwise eligible content.

Videos were classified as containing incorrect or potentially misleading information if they included at least one predefined problematic claim, including product recommendations without indication-based justification, absolute claims regarding warm compresses or eyelid massage, oversimplification of dry eye as a single-cause condition, “one-size-fits-all” treatment advice, statements inconsistent with current guidelines or consensus statements, commercial bias, or exaggerated curative claims such as “radical cure,” “complete cure,” or “cure.” The composite variable “any incorrect or potentially misleading information” was used to indicate whether a video contained at least one such problematic claim, and it was not intended to represent a weighted severity score. Because these categories may differ in clinical severity and implications, each specific category was also recorded separately and reported descriptively.

Incorrect or potentially misleading information was independently coded by two researchers with ophthalmology backgrounds according to the operational criteria listed in Supplementary Table S2. Seven predefined categories were coded as binary variables, including product recommendations without indication-based justification, absolute claims regarding warm compresses or eyelid massage, single-cause explanations, one-size-fits-all treatment advice, statements inconsistent with current guidelines or consensus statements, commercial bias, and exaggerated curative claims. The composite variable “any incorrect or potentially misleading information” was coded as positive if a video contained at least one of these predefined problematic claims. Inter-rater agreement before final adjudication was assessed using Cohen’s kappa and percentage agreement for the composite variable and for each specific category. Disagreements or uncertain cases were resolved through discussion, and unresolved cases were adjudicated by a third researcher before the final analytic dataset was established.

### 2.5 Video quality assessment

All included videos were independently assessed by two researchers with ophthalmology backgrounds. Video quality was evaluated using three commonly used instruments: DISCERN, PEMAT-A/V, and the Global Quality Score (GQS) (31–33). DISCERN was originally developed to assess the reliability and quality of treatment-related written consumer health information, but it has also been widely used in studies evaluating online health information and health-related videos because many videos provide treatment choices, self-management advice, and healthcare-seeking recommendations. In this study, DISCERN was applied to dry eye-related treatment and management information presented through spoken narration, subtitles, on-screen text, and visual materials. PEMAT-A/V was selected because it is specifically designed to assess the understandability and actionability of audiovisual patient education materials. GQS was used as a global assessment of overall information quality, structural completeness, and practical usefulness for patients.

Before formal scoring, the two raters reviewed the original DISCERN, PEMAT-A/V, and GQS scoring instructions and developed a standardized Chinese scoring manual adapted to short-video materials. The manual specified how to evaluate spoken narration, subtitles, on-screen text, images, source transparency, treatment-related claims, product-related statements, risk–benefit descriptions, and action-oriented recommendations. A pilot assessment was conducted using 10 videos before formal scoring. During the pilot assessment, the two raters independently scored the videos, discussed discrepancies, and refined the scoring rules to improve consistency. These pilot videos were subsequently rescored during the formal assessment according to the finalized scoring manual.

During formal scoring, each rater watched the entire video, including audio, subtitles, on-screen text, and images, and recorded item-level scores in a standardized form. Engagement metrics were not included in the scoring form during quality assessment to reduce potential popularity-related bias. The final scores used in the statistical analyses were calculated as the mean scores of the two raters. Inter-rater agreement was evaluated using the intraclass correlation coefficient (ICC) based on a two-way random-effects, absolute-agreement model, with ICC (2, 1), ICC (2, *k*), and their 95% confidence intervals reported. Because the formal statistical analyses were based on the mean scores of the two raters, the main text primarily reports the average-measures ICC, ICC (2, *k*). Because the GQS is an ordinal scale, quadratic-weighted Cohen’s kappa was additionally used as a sensitivity analysis.

### 2.6 Statistical analysis

The Shapiro–Wilk test was used to assess the distribution of continuous variables. As most continuous variables were non-normally distributed, they were summarized as medians and interquartile ranges (IQRs), whereas categorical variables were summarized as counts and percentages. Comparisons between individual medical users and non-medical individual users were performed using the Mann–Whitney *U* test for continuous variables and the *χ*^2^ test or Fisher’s exact test for categorical variables, as appropriate. Associations among video characteristics, the number of covered content domains, and quality scores were assessed using Spearman’s rank correlation analysis. The primary outcomes for interpretation were specified as the DISCERN total score, PEMAT understandability score, GQS total score, and the presence of any incorrect or potentially misleading information. Other uploader-type comparisons, engagement metrics, and correlation analyses were considered exploratory. Because these exploratory analyses were intended to describe patterns rather than to test confirmatory hypotheses, no formal adjustment for multiple comparisons was applied, and the results of these analyses were interpreted cautiously. As a predefined secondary analysis, differences in video duration and engagement metrics between included educational/experience-sharing videos and excluded promotional videos were compared using the Mann–Whitney *U* test. This secondary analysis was conducted only to describe dissemination and engagement characteristics and was not part of the main quality assessment. For sparse binary outcomes, Fisher’s exact test was used, and a Haldane-Anscombe correction was performed as a sensitivity analysis by adding 0.5 to each cell of the 2 × 2 table. Inter-rater agreement for uploader classification and for incorrect or potentially misleading information coding was assessed using Cohen’s kappa. Percentage agreement was also reported for incorrect or potentially misleading information because some specific categories had low prevalence. The 95% confidence intervals for ICCs and Cohen’s kappa values were estimated using 5,000 bootstrap resamples. All analyses were conducted using Python 3.13 with pandas, SciPy, and statsmodels. All statistical tests were two-sided, and *p* < 0.05 was considered statistically significant.

## 3 Results

### 3.1 Video screening process and basic characteristics of the included videos

On April 16, 2026, we searched the “Video” section of Xiaohongshu using the Chinese keyword “干眼” (dry eye), and initially retrieved 200 videos based on the platform’s default sorting algorithm. After screening, 64 videos were excluded, including 41 promotional videos, 16 off-topic videos, 4 items of reposted content, and 3 duplicates, leaving 136 videos for subsequent analysis.

**Figure 1:**
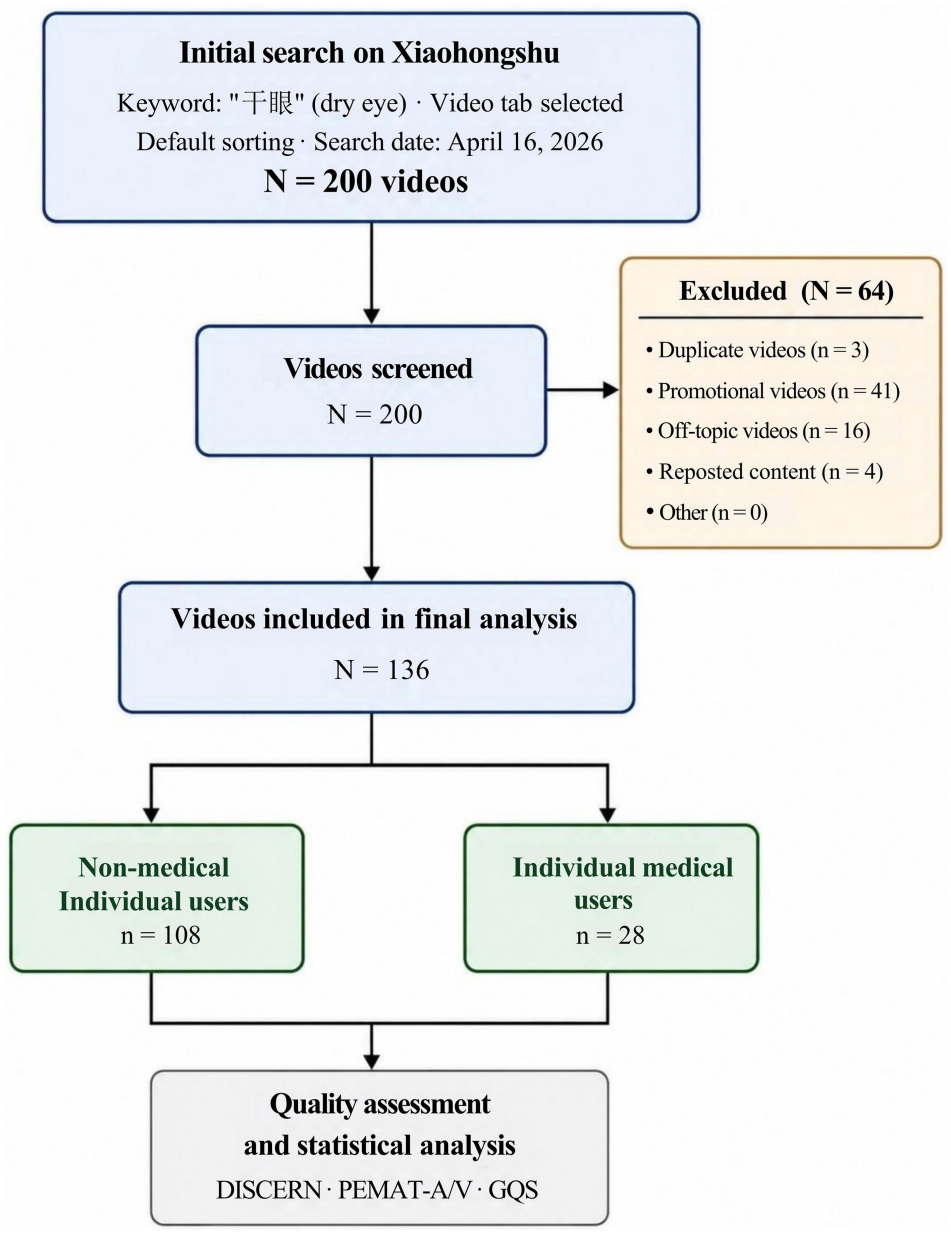
Flowchart of the screening process for dry eye-related videos on Xiaohongshu. On April 16, 2026, the Xiaohongshu “Video” section was searched using the Chinese keyword “干眼” (dry eye) under the default sorting algorithm, yielding 200 videos initially. After excluding 3 duplicates, 41 promotional videos, 16 off-topic videos, and 4 items of reposted content, 136 videos were included in the final analysis. Of these, 108 were uploaded by non-medical individual users and 28 by individual medical users. All included videos were evaluated using DISCERN, PEMAT-A/V, and GQS and were included in subsequent statistical analyses.

Among the included videos, 108 were uploaded by non-medical individual users and 28 by individual medical users. The two reviewers agreed on 134 of 136 uploader classifications. Cohen’s *κ* for uploader classification was 0.954 (95% CI: 0.880–1.000). The two discrepant cases were resolved through discussion or adjudication by a third reviewer before the final analytic dataset was established. The final analytic sample included only two types of individual accounts, as content from for-profit organizations or institutions was mainly excluded during screening because of its promotional nature. All included videos were then assessed using DISCERN, PEMAT-A/V, and GQS and entered into subsequent statistical analyses (Figure 1).

Among the 136 included dry eye-related videos on Xiaohongshu, most were uploaded by non-medical individual users (108/136, 79.4%), whereas 28 videos (20.6%) were uploaded by individual medical users. Significant differences were observed between the two uploader groups across several variables. Compared with videos from non-medical individual users, those from individual medical users had a shorter video duration, received more shares, and covered more content domains, with median values of 73 (56.8, 133.5) s and 134 (91, 217.2) s, respectively (*p* < 0.001), 384.5 (117.5, 1324.2) and 99 (25, 497), respectively (*p* = 0.007), and 3.5 (2, 5) and 2 (1, 3), respectively (*p* < 0.001). The proportions of verified accounts and doctor appearance were also markedly higher among videos from individual medical users, both reaching 92.9%, compared with 1.9 and 0.0%, respectively, among videos from non-medical individual users (both *p* < 0.001). Incorrect or potentially misleading information was more frequent in videos from non-medical individual users than in those from individual medical users (57.4% vs. 10.7%; OR = 11.23, 95% CI: 3.20–39.47; Fisher’s exact test, *p* = 9.93 × 10^−6^). No statistically significant differences were found between the two groups in likes (*p* = 0.397), comments (*p* = 0.663), saves (*p* = 0.600), days from posting to search (*p* = 0.175), product recommendation (*p* = 0.438), or brand visibility (*p* = 0.101) (Table 1).

**Table 1:** Basic characteristics of Xiaohongshu videos related to dry eye disease by uploader type.

Variable	Overall (*N* = 136)	Non-medical individual users (*n* = 108)	Individual medical users (*n* = 28)	*p*-value
Video duration (s)	125 (72, 186.2)	134 (91, 217.2)	73 (56.8, 133.5)	<0.001
Likes	1046.5 (335, 4,574)	929.5 (310, 4,574)	1,395 (503, 4637.8)	0.397
Comments	37 (19, 110.2)	36 (19, 111.5)	46.5 (20.5, 106.5)	0.663
Saves	838.5 (207.8, 3890.2)	758.5 (184.2, 3797.2)	1,156 (299.5, 3898.8)	0.600
Shares	132.5 (28.5, 762.2)	99 (25, 497)	384.5 (117.5, 1324.2)	0.007
Days from posting to search	208 (61.2, 381.2)	204 (47.2, 361.8)	274.5 (148.2, 421.5)	0.175
Number of content domains covered	2 (1, 3)	2 (1, 3)	3.5 (2, 5)	<0.001
Verified account, *n* (%)	28 (20.6%)	2 (1.9%)	26 (92.9%)	<0.001
Doctor appearing on camera, *n* (%)	26 (19.1%)	0 (0.0%)	26 (92.9%)	<0.001
Product recommendation, *n* (%)	72 (52.9%)	59 (54.6%)	13 (46.4%)	0.438
Brand visibility, *n* (%)	36 (26.5%)	32 (29.6%)	4 (14.3%)	0.101
Any incorrect or potentially misleading information, *n* (%)	65 (47.8%)	62 (57.4%)	3 (10.7%)	<0.001

Values are presented as median (IQR) or *n* (%). *p*-values were calculated using the Mann–Whitney *U* test, *χ*^2^ test, or Fisher’s exact test, as appropriate. Verified account refers to the presence of an official platform verification label and was coded separately from uploader type.

### 3.2 Characteristics of video content coverage

**Figure 2:**
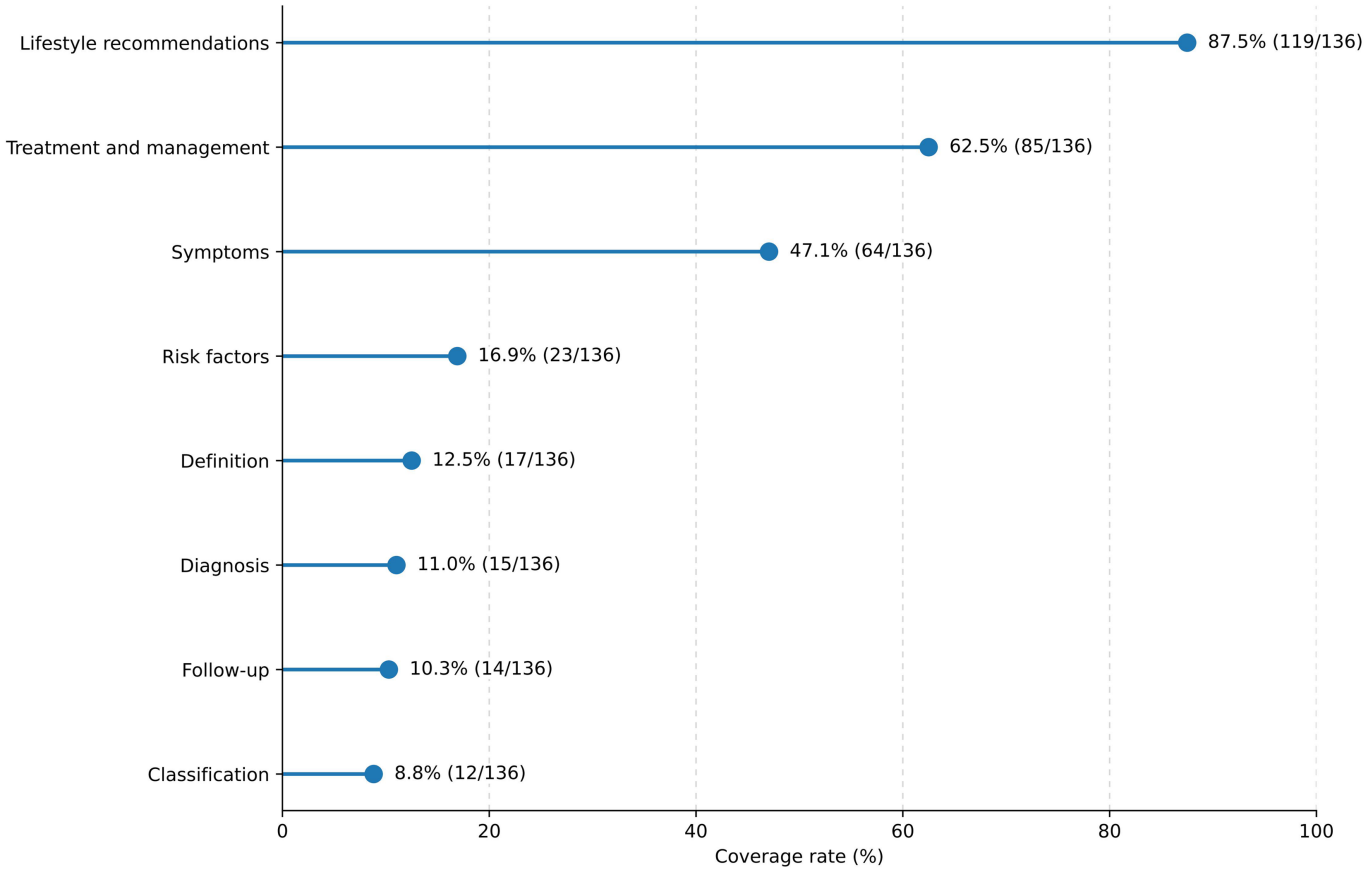
Coverage of content domains in dry eye-related videos on Xiaohongshu. Among the 136 included dry eye-related videos on Xiaohongshu, lifestyle recommendations were most common, with a coverage rate of 87.5% (119/136), followed by treatment and management (62.5%, 85/136), and symptoms (47.1%, 64/136). Risk factors, definition, diagnosis, follow-up, and classification were less frequently covered, with rates of 16.9% (23/136), 12.5% (17/136), 11.0% (15/136), 10.3% (14/136), and 8.8% (12/136), respectively.

The 136 included dry eye-related videos on Xiaohongshu showed an uneven distribution of content domains. The median number of content domains covered per video was 2 (IQR 1–3). Lifestyle recommendations were the most frequently covered domain, appearing in 87.5% of videos (119/136), followed by treatment and management in 62.5% (85/136), while dry eye symptoms were mentioned in 47.1% (64/136). In contrast, content addressing basic disease knowledge and standardized medical information was less frequently presented. Risk factors, definition, and diagnosis were covered in only 16.9% (23/136), 12.5% (17/136), and 11.0% (15/136) of videos, respectively, while follow-up and classification were even less common, appearing in 10.3% (14/136) and 8.8% (12/136), respectively (Figure 2).

### 3.3 Inter-rater agreement

The two raters demonstrated moderate to excellent agreement across the scoring instruments. For single-measure agreement, the ICC (2, 1) values for the DISCERN total score, PEMAT understandability, PEMAT actionability, and GQS total score were 0.974 (95% CI: 0.966–0.980), 0.876 (95% CI: 0.826–0.910), 0.974 (95% CI: 0.933–1.000), and 0.638 (95% CI: 0.541–0.714), respectively. The corresponding average-measure ICC (2, *k*) values were 0.987 (95% CI: 0.983–0.990), 0.934 (95% CI: 0.905–0.953), 0.987 (95% CI: 0.965–1.000), and 0.779 (95% CI: 0.702–0.833), respectively. Sensitivity analysis for the ordinal GQS yielded a quadratic-weighted Cohen’s *κ* of 0.636 (95% CI: 0.548–0.724), supporting the robustness of the agreement assessment.

Inter-rater agreement was assessed for the coding of incorrect or potentially misleading information. For the composite variable “any incorrect or potentially misleading information,” agreement was 96.3% (131/136), and Cohen’s *κ* was 0.926 (95% CI: 0.854–0.985). For the seven specific categories of incorrect or potentially misleading information, percentage agreement ranged from 97.8 to 100.0%, and Cohen’s *κ* values ranged from 0.933 to 1.000. Disagreements were resolved through discussion or adjudication by a third researcher, and the final adjudicated dataset was used for subsequent analyses.

### 3.4 Comparison of video quality scores by uploader type

Significant differences were observed across all quality assessment metrics between dry eye-related videos uploaded by different uploader types on Xiaohongshu (Table 2, all *p* < 0.001). Compared with videos from non-medical individual users, those from individual medical users had higher DISCERN total scores, reliability subscale scores, treatment choice subscale scores, and overall quality ratings; the median DISCERN total scores were 58 (53.2, 62.2) and 35.5 (31.4, 40.1), respectively. Videos from individual medical users also had higher PEMAT understandability scores than those from non-medical individual users, with median scores of 100 (95.4, 100) and 77.1 (65, 87.7), respectively. Although the median PEMAT actionability score was 100 in both groups, scores among videos from individual medical users were more concentrated at the upper end, with median scores of 100 (100, 100) and 100 (66.7, 100), respectively. The GQS total score was also substantially higher among videos from individual medical users than among those from non-medical individual users, with median scores of 4.5 (4.5, 4.5) and 3.0 (2.5, 3.5), respectively.

**Table 2:** Quality assessment scores of dry eye-related Xiaohongshu videos by uploader type.

Variable	Overall (*N* = 136)	Non-medical individual users (*n* = 108)	Individual medical users (*n* = 28)	*p*-value
DISCERN total score	37 (31.5, 48.8)	35.5 (31.4, 40.1)	58 (53.2, 62.2)	<0.001
DISCERN reliability subscale	19.5 (16.9, 27)	18.5 (16, 21.5)	30.8 (30, 32.5)	<0.001
DISCERN treatment choice subscale	14.5 (11.5, 18.5)	14 (11, 16.1)	22.5 (18.9, 27)	<0.001
DISCERN overall quality rating	3.2 (2.5, 4)	3 (2.5, 3.5)	4.5 (4, 5)	<0.001
PEMAT understandability score	80.4 (68.2, 95.5)	77.1 (65, 87.7)	100 (95.4, 100)	<0.001
PEMAT actionability score	100 (66.7, 100)	100 (66.7, 100)	100 (100, 100)	<0.001
GQS total score	3.5 (3, 4)	3 (2.5, 3.5)	4.5 (4.5, 4.5)	<0.001

Values are presented as median (IQR). *p*-values were calculated using the Mann–Whitney *U* test.

**Figure 3:**
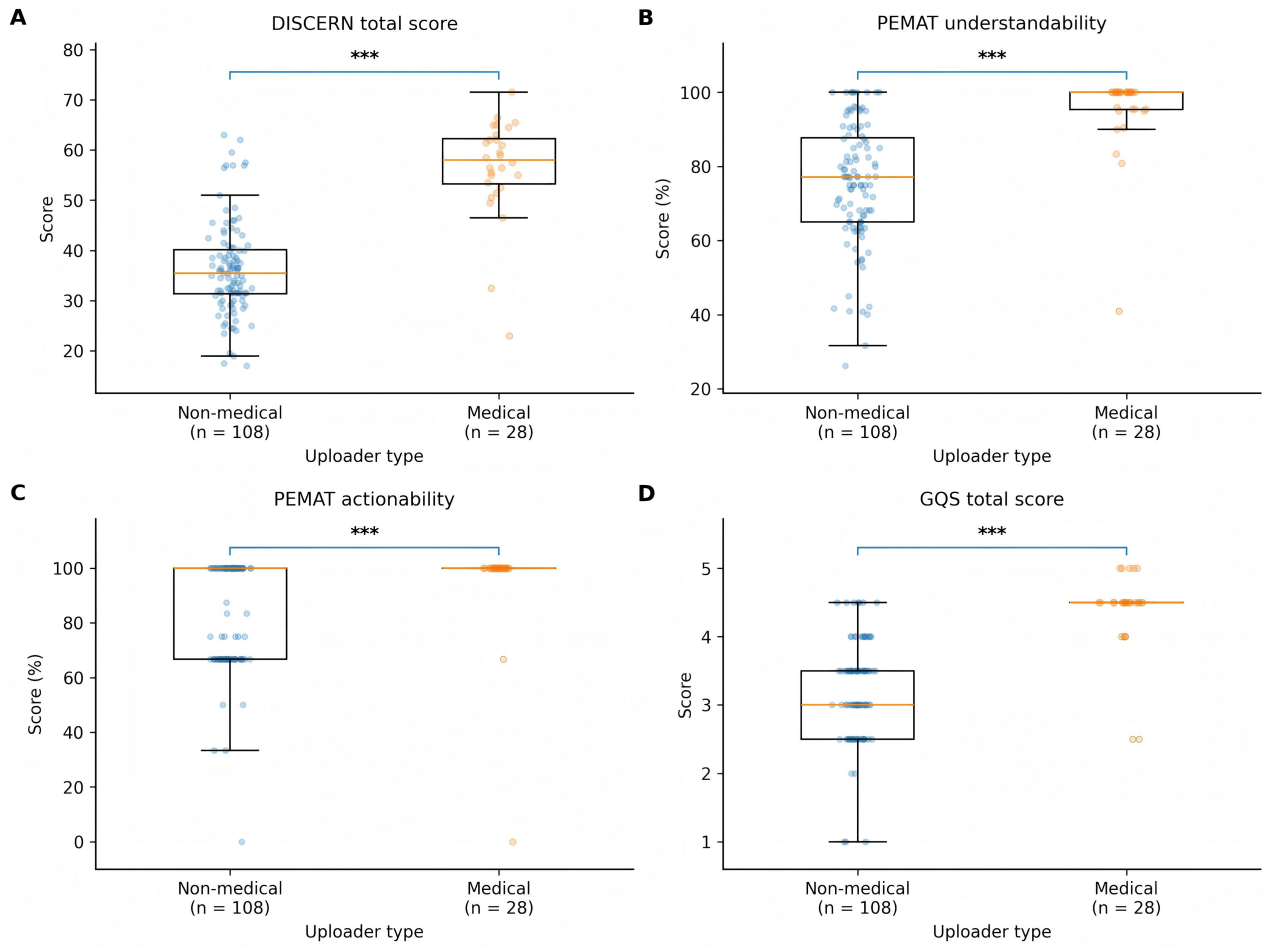
Comparison of quality assessment scores for dry eye-related videos on Xiaohongshu by uploader type. **(A)** DISCERN total score; **(B)** PEMAT understandability score; **(C)** PEMAT actionability score; **(D)** GQS total score. Boxplots show medians and interquartile ranges, with dots representing individual videos. For readability, “Non-medical” and “Medical” in the x-axis labels denote non-medical individual users and individual medical users, respectively. Compared with videos from non-medical individual users, those from individual medical users had significantly higher DISCERN total scores, PEMAT understandability scores, PEMAT actionability scores, and GQS total scores (all *p* < 0.001). ***p < 0.001.

Figure 3 further illustrates the distribution of DISCERN total score, PEMAT understandability, PEMAT actionability, and GQS total score by uploader type. Videos uploaded by individual medical users scored higher overall than those from non-medical individual users in DISCERN total score, PEMAT understandability, PEMAT actionability, and GQS total score (all *p* < 0.001).

### 3.5 Correlation analysis among video characteristics, engagement metrics, and quality scores

**Figure 4:**
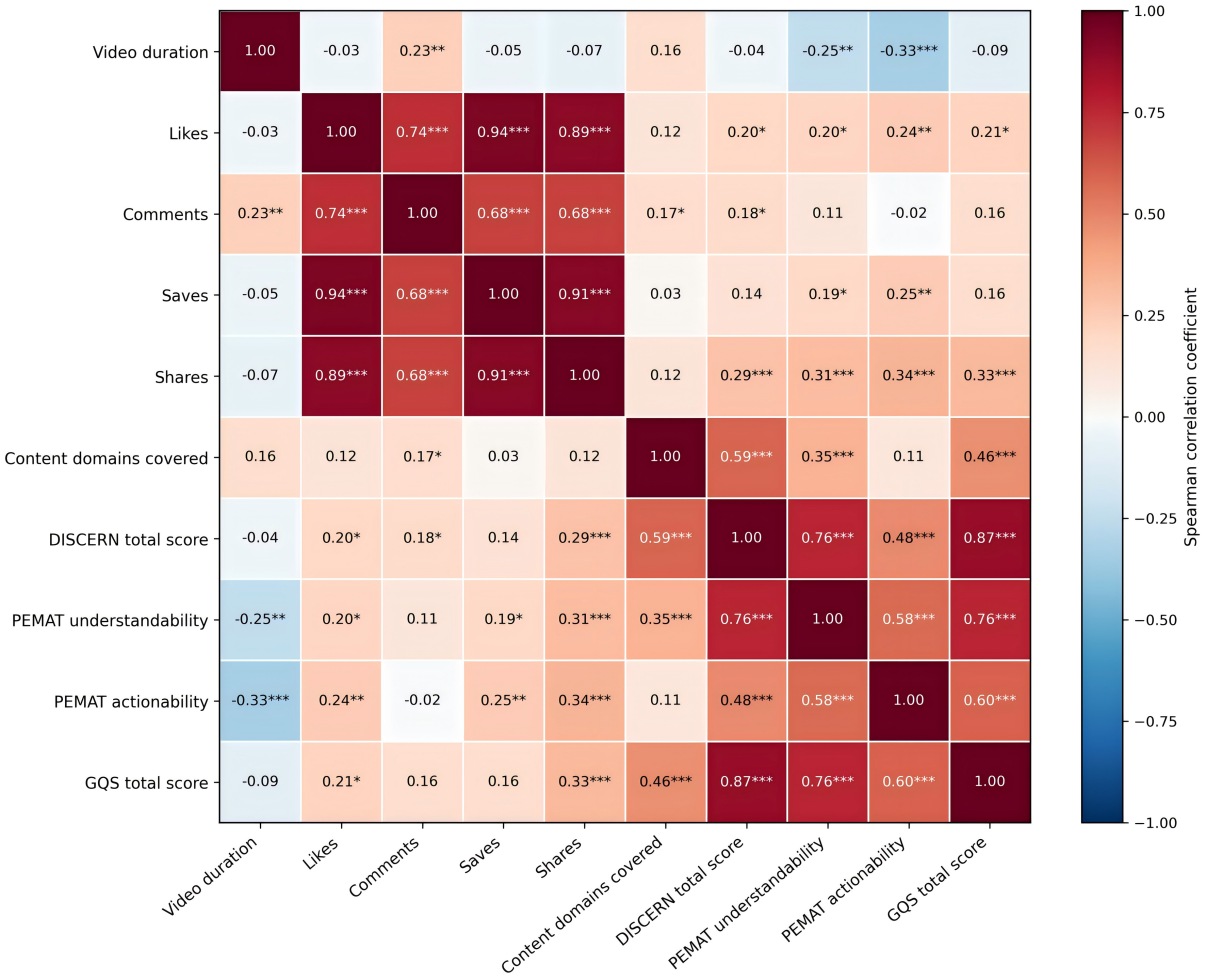
Spearman correlation heatmap among video characteristics, content coverage, and quality scores of dry eye-related videos on Xiaohongshu. The heatmap displays Spearman correlation coefficients among video duration, user engagement metrics (likes, comments, saves, and shares), number of covered content domains, DISCERN total score, PEMAT understandability, PEMAT actionability, and GQS total score. Red indicates positive correlations, blue indicates negative correlations, and darker colors indicate stronger correlation coefficients. Engagement metrics showed strong positive correlations with each other; the number of covered content domains was positively correlated with DISCERN total score, PEMAT understandability, and GQS total score; shares were modestly positively correlated with multiple quality scores; and video duration was negatively correlated with PEMAT understandability and PEMAT actionability. The correlation heatmap was used as an exploratory analysis to describe patterns among variables. *p < 0.05; **p < 0.01; ***p < 0.001.

The correlation analysis was exploratory and was intended to describe patterns among engagement metrics, content coverage, and quality scores. User engagement metrics were generally strongly and positively correlated, with likes showing strong correlations with saves (*r* = 0.94, *p* < 0.001), shares (*r* = 0.89, *p* < 0.001), and comments (*r* = 0.74, *p* < 0.001); saves were also strongly correlated with shares (*r* = 0.91, *p* < 0.001). Among the quality assessment metrics, DISCERN total score was positively correlated with GQS total score (*r* = 0.87, *p* < 0.001), PEMAT understandability (*r* = 0.76, *p* < 0.001), and PEMAT actionability (*r* = 0.48, *p* < 0.001); PEMAT understandability was also positively correlated with GQS total score (*r* = 0.76, *p* < 0.001) and PEMAT actionability (*r* = 0.58, *p* < 0.001) (Figure 4).

Further analysis showed that the number of covered content domains was positively associated with DISCERN total score (*r* = 0.59, *p* < 0.001), GQS total score (*r* = 0.46, *p* < 0.001), and PEMAT understandability (*r* = 0.35, *p* < 0.001). In this exploratory analysis, shares were modestly associated with DISCERN total score (*r* = 0.29, *p* < 0.001), PEMAT understandability (*r* = 0.31, *p* < 0.001), PEMAT actionability (*r* = 0.34, *p* < 0.001), and GQS total score (*r* = 0.33, *p* < 0.001). Video duration was negatively associated with PEMAT understandability (*r* = −0.25, *p* < 0.01) and PEMAT actionability (*r* = −0.33, *p* < 0.001) (Figure 4).

### 3.6 Video content composition and distribution of incorrect or potentially misleading information by uploader type

**Figure 5:**
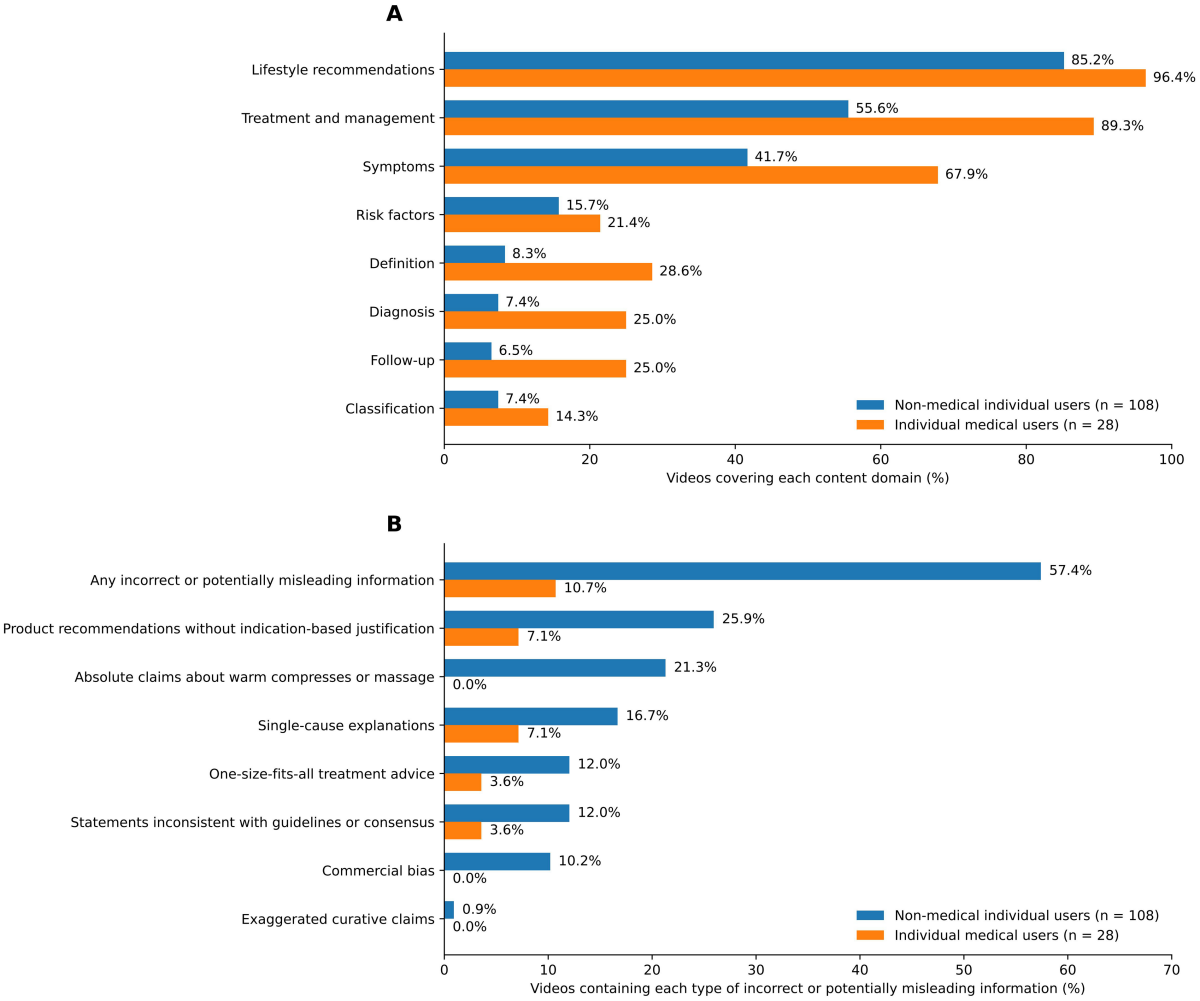
Content coverage and distribution of incorrect or potentially misleading information in dry eye-related videos on Xiaohongshu by uploader type. **(A)** Comparison of content domain coverage between videos from non-medical individual users and individual medical users, including lifestyle recommendations, treatment and management, symptoms, risk factors, definition, diagnosis, follow-up, and classification. Videos from individual medical users showed higher coverage rates in most content domains. **(B)** Distribution of different types of incorrect or potentially misleading information in videos from the two uploader groups, including any incorrect or potentially misleading information, product recommendations without indication-based justification, absolute claims about warm compresses or massage, single-cause explanations, “one-size-fits-all” treatment advice, statements inconsistent with guidelines or consensus, commercial bias, and exaggerated curative claims. Incorrect or potentially misleading information was more frequently observed in videos from non-medical individual users. Blue indicates non-medical individual users, and orange indicates individual medical users.

Dry eye-related videos differed substantially in content coverage across uploader types. Videos in both groups primarily focused on lifestyle recommendations and treatment and management, whereas videos from individual medical users showed higher coverage rates in most content domains. Specifically, videos from individual medical users covered lifestyle recommendations, treatment and management, and symptoms at rates of 96.4, 89.3, and 67.9%, respectively, all higher than the corresponding rates among videos from non-medical individual users (85.2, 55.6, and 41.7%). More systematic medical topics, including definition, diagnosis, follow-up, and classification, were also more frequently presented in videos from individual medical users, suggesting a more complete content structure and a more comprehensive presentation of disease-related knowledge (Figure 5A).

Incorrect or potentially misleading information was more common in videos from non-medical individual users. The prevalence of any incorrect or potentially misleading information was 57.4% in videos from non-medical individual users and 10.7% in videos from individual medical users (62/108 vs. 3/28; OR = 11.23, 95% CI: 3.20–39.47; Fisher’s exact test, *p* = 9.93 × 10^−6^). In the sensitivity analysis using the Haldane-Anscombe correction, the association remained similar, with a corrected OR of 9.79 (95% CI: 3.01–31.87). Specifically, videos from non-medical individual users more frequently contained product recommendations without indication-based justification, absolute claims about warm compresses or massage, single-cause explanations, “one-size-fits-all” treatment advice, statements inconsistent with guidelines or consensus, and commercial bias. These issues were generally less frequent in videos from individual medical users, and no absolute claims about warm compresses or massage, commercial bias, or exaggerated curative claims were observed in this group (Figure 5B).

### 3.7 Comparison of engagement characteristics between included and promotional videos

**Figure 6:**
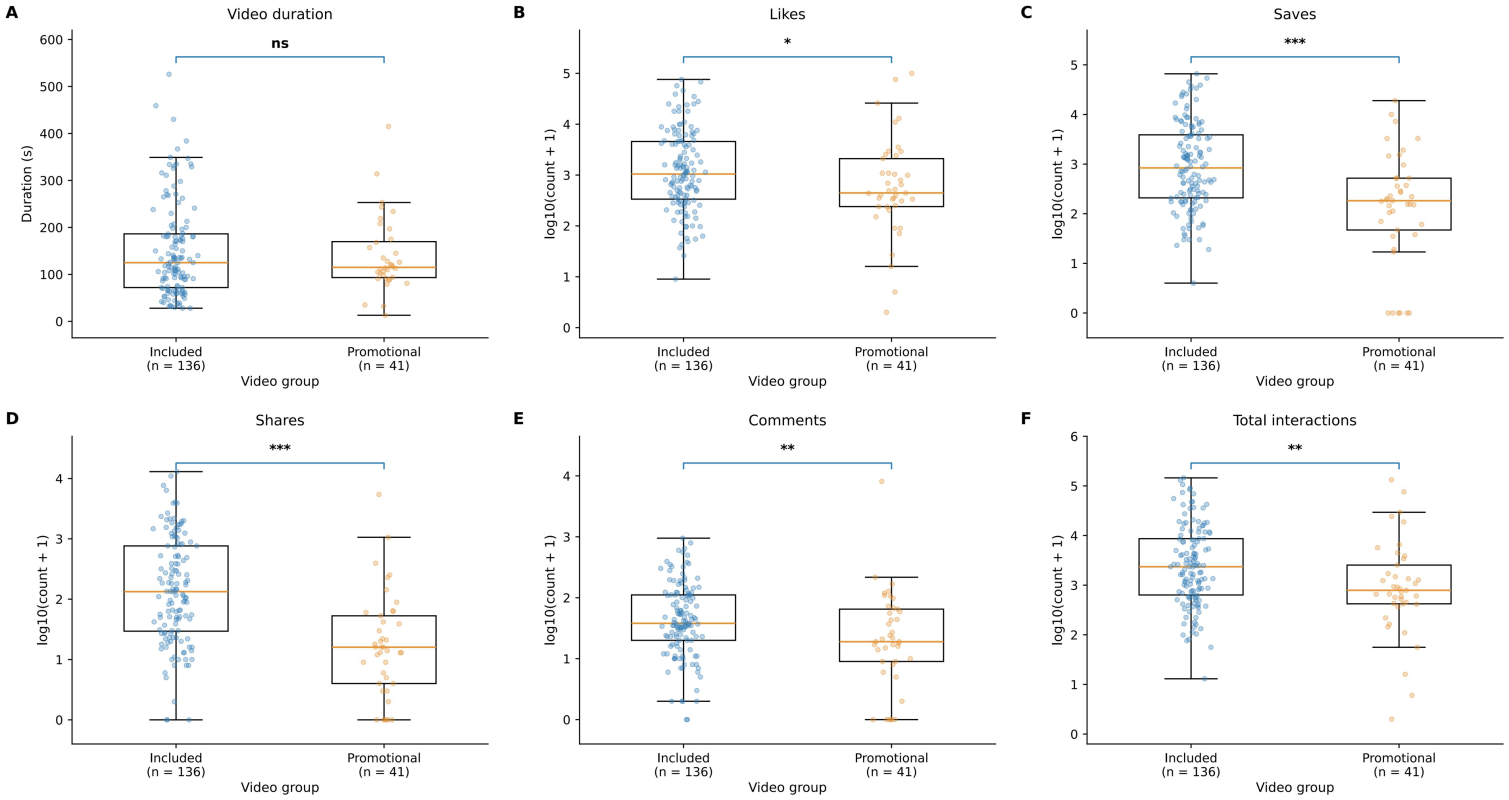
Comparison of video duration and user engagement metrics between included and promotional videos. **(A)** Video duration; **(B)** Likes; **(C)** Saves; **(D)** Shares; **(E)** Comments; **(F)** Total interactions. Except for video duration, all engagement metrics are displayed after log10 (count + 1) transformation. Boxplots show medians and interquartile ranges, with dots representing individual videos. Included videos (*n* = 136) showed higher likes, saves, shares, comments, and total interactions than promotional videos (*n* = 41), while video duration did not differ significantly between the two groups. NS indicates no statistical significance; *p < 0.05; **p < 0.01; ***p < 0.001.

In the predefined secondary descriptive analysis, we compared publicly displayed raw video duration and engagement metrics between the 136 included educational/experience-sharing videos and the 41 excluded promotional videos. No statistically significant difference in video duration was observed between the two groups (*p* > 0.05). In terms of engagement, included videos had higher numbers of likes, saves, shares, comments, and total interactions than promotional videos. The difference was statistically significant for likes (*p* < 0.05), saves and shares (both *p* < 0.001), and comments and total interactions (both *p* < 0.01) (Figure 6). This analysis was descriptive and was not included in the main quality assessment.

## 4 Discussion

In this cross-sectional study, we evaluated the quality, content coverage, and uploader-type differences of dry eye-related educational and experience-sharing videos on Xiaohongshu. We interpreted these findings in the context of Xiaohongshu’s lifestyle- and experience-sharing content ecology, where personal narratives, product-use experiences, and health advice frequently coexist. The findings showed substantial variation in information quality. Video content mainly focused on lifestyle recommendations and treatment management, whereas systematic medical information, including definition, classification, diagnosis, and follow-up, was insufficiently covered. In this sample, videos uploaded by individual medical users had broader content coverage, a lower prevalence of incorrect or potentially misleading information, and higher DISCERN, PEMAT-A/V, and GQS scores than those uploaded by non-medical individual users. Broader content coverage was associated with higher reliability, understandability, and overall quality scores. Shares were also modestly associated with several quality scores, although this association should be interpreted cautiously because engagement metrics may be influenced by account visibility, posting time, follower base, and platform recommendation algorithms. These exploratory associations should be interpreted as descriptive findings rather than confirmatory evidence.

The platform-specific findings may be partly explained by the experience-sharing ecology of Xiaohongshu. Personal narratives are often relatable and may facilitate engagement, but they may also transform individual experiences into generalized health recommendations. This issue is particularly relevant to dry eye because dry eye is a multifactorial disease that requires individualized assessment and long-term management. Advice on artificial tears, warm compresses, eyelid massage, or eye-care products may be useful for some users, but may become misleading when presented as universally applicable, curative, or not requiring indication-based judgment. Although inter-rater agreement for this coding framework was high, the assessment of incorrect or potentially misleading information still involved clinical judgment, especially when distinguishing neutral product-use descriptions from product recommendations without indication-based justification or commercial bias. The composite variable should therefore be interpreted as the presence of at least one problematic claim rather than as a weighted severity measure. Taken together, Xiaohongshu provides a distinctive context for examining how lifestyle narratives and product-oriented content intersect with ophthalmic health education.

These findings are consistent with the general trends reported in previous studies of health-related short videos. Huang et al. (20) found that dry eye-related videos on Douyin mainly focused on symptoms, risk factors, and management recommendations, with limited coverage of systematic knowledge such as definition, diagnosis, and classification. This pattern is similar to our observations on Xiaohongshu. Previous studies of short videos on age-related macular degeneration, stroke, pediatric influenza vaccination, Crohn’s disease, thyroid-associated ophthalmopathy, ankle sprain, and congenital nasolacrimal duct obstruction have also reported clear quality differences across platforms and uploader sources. Several of these studies found that videos from professional or institutional sources performed better in reliability, understandability, or overall quality (21–26, 34). These findings suggest that uploader expertise and platform content ecology may be associated with the scientific accuracy and actionability of health information in short videos. Notably, non-medical individual users accounted for the majority of uploaders in this study and showed a substantially higher prevalence of incorrect or potentially misleading information, a finding that differs from some studies primarily based on Douyin or Bilibili (20–25). However, this effect estimate should be interpreted cautiously because the individual medical user group was relatively small and only three videos in this group contained incorrect or potentially misleading information. This difference further suggests that uploader composition and platform-specific content norms should be considered when comparing health-information quality across Chinese short-video platforms.

Compared with many previous studies that emphasized multi-platform comparisons or classified uploaders into broad source categories, this study focused on the “Video” section of Xiaohongshu and specifically examined differences between individual medical users and non-medical individual users, two uploader groups that are highly relevant to this platform environment. In addition to using DISCERN, PEMAT-A/V, and GQS, we developed a dry eye-specific framework for assessing content coverage and identifying incorrect or potentially misleading information (31–33). This design allowed us to simultaneously assess whether the content was comprehensive, whether the information was understandable, whether the recommendations were actionable, and where incorrect or potentially misleading information was most likely to occur. In this study, videos from individual medical users were shorter, covered more content domains, and had a lower prevalence of incorrect or potentially misleading information. They also received more shares in the descriptive comparison, but this finding should be interpreted cautiously because engagement metrics may be influenced by account visibility, follower base, posting time, and platform recommendation algorithms. For chronic ocular surface diseases such as dry eye, these findings suggest that professionally curated short videos may provide educational value, although their actual dissemination effects and behavioral impact require further investigation. The combined use of multidimensional assessment instruments and disease-specific content analysis has also been increasingly applied in studies of dry eye-related and other health-related short videos (29, 30).

This study has several limitations. First, this was a single-time-point cross-sectional analysis based on the platform’s default sorting algorithm and a single search keyword, which may have introduced selection bias toward videos with greater platform exposure and may have missed relevant videos retrieved by related Chinese search terms, such as “干眼症” (dry eye disease), “眼干” (eye dryness), “眼睛干涩” (ocular dryness), “人工泪液” (artificial tears), “睑板腺功能障碍” (meibomian gland dysfunction), or “MGD.” Although we used a newly registered account without prior browsing or search history to minimize prior personalization, Xiaohongshu’s ranking algorithm is proprietary and dynamic, and search results may still be influenced by geographic location, device environment, search time, and platform-level recommendation mechanisms. In addition, repeated searches were not performed, and excluding videos primarily intended for advertising or product sales may have underestimated commercial bias and product-oriented incorrect or potentially misleading information in the overall platform environment. Second, this study included only Chinese-language videos from Xiaohongshu, and the sample size of individual medical users was relatively limited; therefore, the generalizability of the findings to other platforms, languages, and ophthalmic topics should be interpreted with caution. In addition, uploader type overlapped with several video- and account-level characteristics, including account verification, doctor appearance on camera, video duration, content coverage, product-related information, time since posting, and potential algorithmic exposure. Therefore, the observed uploader-type differences should be interpreted as associations rather than independent effects of medical identity itself. Third, although two trained raters independently conducted content coding and quality assessment and inter-rater agreement was evaluated, manual assessment may still involve some degree of subjective judgment. In particular, the single-measure ICC and quadratic-weighted kappa for GQS indicated only moderate agreement, suggesting greater subjectivity than DISCERN and PEMAT-A/V. Therefore, GQS findings were interpreted together with the other quality assessment tools rather than as standalone evidence. Viewer demographics, comment semantics, viewing completion rates, platform recommendation pathways, and behavioral outcomes were not available. Therefore, the observed correlations should not be interpreted as causal relationships. Future studies could conduct multi-keyword and multi-platform comparisons across different ophthalmic topics over longer time periods and incorporate additional indicators, such as comment semantics, viewing completion rates, interaction pathways, and behavioral outcomes, to further evaluate the real-world impact of short-video health communication. In addition, more ophthalmology-specific and platform-adapted evaluation frameworks could be developed for dry eye and other common ocular surface diseases, with further integration into broader research on the credibility of health content on Chinese short-video platforms (35), video large language model-based assessment and AI-supported ophthalmic education (36, 37), disease-specific AI applications and broader ophthalmology-specific evidence frameworks (38–43), and ophthalmology-oriented large language model applications and prospects (44).

## 5 Conclusion

This study shows substantial heterogeneity in the information quality of dry eye-related videos on Xiaohongshu. The content was mainly oriented toward lifestyle recommendations and treatment management, whereas systematic medical information, including definition, classification, diagnosis, and follow-up, was insufficiently covered. In this cross-sectional sample, videos uploaded by individual medical users were associated with broader content coverage, higher quality scores, and a lower prevalence of incorrect or potentially misleading information than videos from non-medical individual users. These findings suggest the potential value of platform-level quality control, improved mechanisms for identifying and recommending professional content, and greater participation of ophthalmic healthcare professionals in standardized science communication. Future research should conduct longitudinal comparisons across multiple platforms, time points, and search keywords, while incorporating comment semantics, dissemination pathways, and user behavioral outcomes to further evaluate the real-world impact of short-video health communication and the effectiveness of potential intervention strategies.

## Data Availability

The raw data supporting the conclusions of this article will be made available by the authors, without undue reservation.
