# Supplementary material for: Quality assessment of dry eye educational videos on Xiaohongshu and differences by uploader type
Source: Front Med (Lausanne). 2026 Jun 3;13:1868829. doi: 10.3389/fmed.2026.1868829 (PMC13272041; doi:10.3389/fmed.2026.1868829)
Supplement: Supplementary file 1 [file Table_1.DOCX]

**Supplementary Table S1. Criteria for uploader classification**

| **Category** | **Operational definition** | **Required or supporting evidence** | **Classification rule** |
| --- | --- | --- | --- |
| Individual medical users | Individual accounts with clearly displayed platform-based medical professional qualification verification on the account profile or clear evidence of medical professional identity based on publicly available account information and video content. | Evidence included platform-based medical professional qualification verification, certified professional category, affiliated institution, professional title or position when available, doctor appearance on camera supported by other publicly available profile information, or consistent provision of specialty-specific medical explanations. | Accounts were classified as individual medical users when they had visible platform-based medical professional qualification verification or sufficient publicly available evidence supporting medical professional identity. Doctor appearance on camera, self-reported medical identity, or health-related content alone was not considered sufficient unless supported by other publicly available profile information or consistent specialty-specific medical explanations. |
| Non-medical individual users | Individual accounts without sufficient evidence of medical professional identity. | Patient experience sharing, lifestyle-oriented content, personal product-use experience, dry eye symptom description, or general health-related content without sufficient evidence of medical professional identity. | Accounts were classified as non-medical individual users when sufficient evidence of medical professional identity was not available, even if they discussed dry eye-related symptoms, products, self-management experiences, or general health topics. |
| Ambiguous accounts | Accounts for which medical identity or account type could not be clearly determined from publicly available profile information and video content. | Examples included self-reported medical identity without supporting evidence, doctor appearance on camera without additional supporting information, medical students, healthcare-related commercial accounts, medical-aesthetic accounts, institutional operation accounts, product-promotion accounts, or accounts with incomplete or inconsistent identity information. | Ambiguous accounts were independently reviewed by two reviewers according to the predefined coding rule. Accounts were classified as individual medical users only when sufficient evidence of medical professional identity was available. Promotional accounts were excluded from the main quality assessment if their primary purpose was advertising or product sales. Disagreements were resolved through discussion or adjudication by a third reviewer. |

Note: Uploader classification was based on publicly available account information and video content. Platform-based medical professional qualification verification was considered the strongest evidence of medical professional identity. The variable “verified account” referred to the presence of an official platform verification label and was coded separately from uploader type.
