# Supplementary material for: Quality assessment of dry eye educational videos on Xiaohongshu and differences by uploader type
Source: Front Med (Lausanne). 2026 Jun 3;13:1868829. doi: 10.3389/fmed.2026.1868829 (PMC13272041; doi:10.3389/fmed.2026.1868829)
Supplement: Supplementary file 2 [file Table_2.DOCX]

**Supplementary Table S2. Operational criteria for incorrect or potentially misleading information**

| **Category** | **Operational definition** | **Judgment boundary** |
| --- | --- | --- |
| Product recommendations without indication-based justification | A video recommended a product category, specific product, or brand for dry eye-related symptoms without explaining applicable indications, suitable users, limitations, potential risks, or the need for clinical evaluation when appropriate. | Neutral mention of a product category was not coded as positive if it was accompanied by indication-based explanation or appropriate caution. |
| Absolute claims about warm compresses or eyelid massage | A video described warm compresses or eyelid massage as universally applicable, necessary for all dry eye patients, or able to solve dry eye without considering dry eye subtype, meibomian gland dysfunction status, inflammation, contraindications, or individual differences. | Advice was not coded as positive when warm compresses or eyelid massage were presented as potentially helpful for selected patients with appropriate caveats. |
| Single-cause explanation | A video attributed dry eye exclusively or predominantly to one cause, such as screen use, staying up late, lack of sleep, or “lack of water,” while ignoring the multifactorial nature of dry eye. | Mentioning one risk factor was not coded as positive if it was presented as one of multiple possible contributors. |
| One-size-fits-all treatment advice | A video provided a fixed treatment or self-management regimen as suitable for all dry eye patients without recommending individualized assessment, symptom-based judgment, or medical consultation when needed. | General lifestyle recommendations were not coded as positive if they were presented as supportive rather than universally sufficient treatment. |
| Statements inconsistent with guidelines or consensus | A video contained statements clearly inconsistent with current dry eye consensus statements or clinical frameworks, including inappropriate treatment recommendations, misleading diagnostic claims, or advice that could delay appropriate medical care. | Simplified explanations were not coded as positive when they remained broadly consistent with current consensus and did not mislead users about diagnosis or treatment. |
| Commercial bias | A video’s health recommendation was strongly oriented toward product purchase, brand promotion, or service use, with commercial messaging outweighing balanced medical explanation. | Incidental brand visibility or product mention was not coded as commercial bias unless the health message was clearly shaped by promotional intent. |
| Exaggerated curative claims | A video claimed or implied that dry eye could be “cured,” “radically cured,” “permanently cured,” or prevented from recurring through a specific product, method, or regimen. | Claims of symptom relief or improvement were not coded as positive when they did not imply cure or permanent resolution. |

Note: Categories were coded separately. When a video contained one or more categories, it was also coded as positive for the composite variable “any incorrect or potentially misleading information.”
